# Current and Clinically Relevant Echocardiographic Parameters to Analyze Left Atrial Function

**DOI:** 10.3390/jcdd11080241

**Published:** 2024-08-05

**Authors:** Mario Mangia, Emilio D’Andrea, Antonella Cecchetto, Riccardo Beccari, Donato Mele, Stefano Nistri

**Affiliations:** 1 Department of Cardiac Thoracic Vascular Sciences and Public Health, University of Padua, 35128 Padova, Italy; mario.mangia@studenti.unipd.it (M.M.); dandreaemilio@gmail.com (E.D.); antonella.cecchetto@aopd.veneto.it (A.C.); riccardo.beccari@studenti.unipd.it (R.B.); donato.mele@unipd.it (D.M.); 2 Cardiology Service, CMSR Veneto Medica, 36077 Altavilla Vicentina, Italy

**Keywords:** cardiovascular imaging, echocardiography, diastolic dysfunction, LAEF, LA strain, TDI-a’, LAVI/a’

## Abstract

The evaluation of the left atrial (LA) size using the LA volume index (LAVI) is clinically relevant due to its prognostic significance in various conditions. Nonetheless, adding a LA function assessment to the LAVI provides further clinical and prognostic information in different cardiovascular (CV) diseases. The assessment of LA function by echocardiography primarily includes volumetric measurements (LA ejection fraction [LAEF]), tissue Doppler imaging (TDI) (mitral annular late diastolic velocity [a’]), and speckle-tracking methods, such as LA longitudinal reservoir strain (LA strain). This review analyzes and discusses the current medical evidence and potential clinical usefulness of these different methods to analyze LA function.

## 1. Introduction

The evaluation of the left atrial (LA) size is key in any comprehensive transthoracic echocardiogram due to its prognostic value in different clinical conditions [1,2,3,4]. To date, the LA volume indexed for body surface area (LAVI) is preferred over the anteroposterior diameter, representing the most accurate measure of LA size by standard transthoracic echocardiography. Since the LA enlarges in the setting of increased left ventricular (LV) filling pressures, the LAVI is considered a good marker of diastolic dysfunction (DD) and is strongly associated with cardiovascular (CV) events [4,5,6,7,8,9,10,11,12]. However, the LAVI does not necessarily mirror DD [13]. Indeed, we [14] and others [15,16] reported an upper limit of a normal LAVI in non-athletes, exceeding that suggested by guidelines [5]. Moreover, discrepancies have been reported among different methods to assess the LAVI [17]. Hence, both for clinical and research purposes, different echocardiographic methods cannot be used interchangeably for diagnosis and follow-up.

An assessment of LA functional parameters provides early insights into pathological changes, offering additional information as compared to the LAVI [7]. Different methods are used to assess LA function as follows: 2D and 3D volume-based approaches, transmitral and pulmonary veins spectral Doppler imaging, tissue Doppler imaging (TDI), strain and strain rates by TDI, and speckle-tracking echocardiography (STE) [8,9]. However, the methods used for estimating LA function may be unpractical in everyday clinical activities, either due to the need for multiple measurements (beyond the LAVI) or to the dependence on the specific software available on dedicated workstations or board-only last-generation echocardiographic machines.

As mitral annular velocities are routinely measured by pulsed-wave TDI as a component of the assessment of diastolic function [18], the peak late diastolic velocity (TDI-a’) might be a candidate to assess LA function in everyday clinical practice [10,11] This review aims to summarize the updated knowledge on TDI-a’, exploring its potential role for evaluating LA function.

## 2. Left Atrial Function: An Introduction

**Figure 1:**
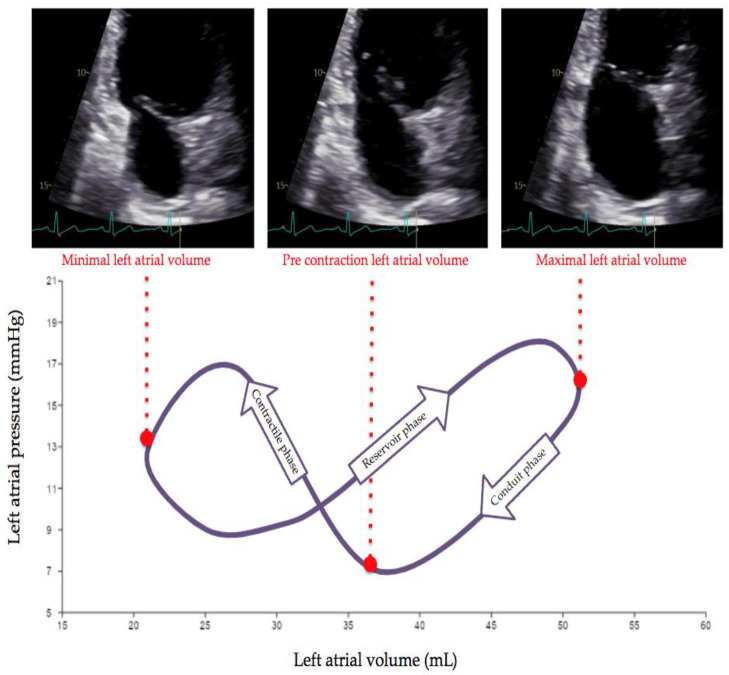
Left atrial pressure-volume loop, with a qualitative representation of a left atrial (LA) pressure-volume loop and corresponding echocardiographic evaluations of LA volumes. Maximal LA volume (usually reported in clinical echocardiography as LAVI) occurs at the end of the reservoir phase, while the conduit phase can be captured by LA volume pre-A. Minimum LA volume corresponds to the end of the LA contractile phase. Superimposed arrows indicate the time-course.

The LA plays a key role in maintaining optimal cardiac performance by dynamically modulating LV filling. The interplay between LA and LV performance is characterized by the three following phases (Figure 1): (a) a “reservoir” phase, when blood moves from the pulmonary veins to the LA, achieving maximum volume (LAVI max); (b) a “conduit” phase in which blood passively shifts from the LA directly into the LV, leaving in the LA a “pre-atrial contraction” volume (LA pre-A); (c) a “booster pump” phase in which the LA actively shifts blood in the LV, reaching a minimum LA volume (LAVI min).

Studies of LA function provide new insights into the pathophysiologic and prognostic role of the LA in CV disease and outcomes [4,5,6,7,8,9], suggesting that LA functional measurements may be superior to the LA size in LV DD and can even precede volumetric changes [19,20,21]. LA function has been historically assessed by a variety of techniques, including time-honored systo-diastolic variations in M-mode LA tracings. Subsequently, volumetric approaches (first by 2D, more recently by 3D techniques) have been used to assess LA function alone (Table 1) [8] or in association with the LV outflow-tract velocity time integral (VTI)—the so-called LA function index (LAFI) [22].

An analysis of pulsed-wave Doppler flow velocities, recorded in the pulmonary veins (PVF) (LA filling) and mitral valve tips (LA emptying), may provide some estimation of LA function (Table 2).

The ratio of peak transmitral early (E) and late (A) transmitral velocities, or velocity time integral (VTI) and the atrial filling fraction (A-VTI/(E-VTI+A-VTI)), estimate the relative contribution of LA contraction to global LA function [23]. The ratio of systolic (S) to diastolic (D) PVF waves estimates the relative contribution of the reservoir to conduit function [24]. The extent and duration of the reversed pulmonary flow wave during atrial contraction (A_r_) is used to estimate LA contraction. LA ejection force [25,26] and LA kinetic energy are used as markers of LA performance. The interpretation of transmitral and PVF patterns is, however, affected by the load conditions and heart rate, age, and LV diastolic properties [27,28,29].

**Figure 2:**
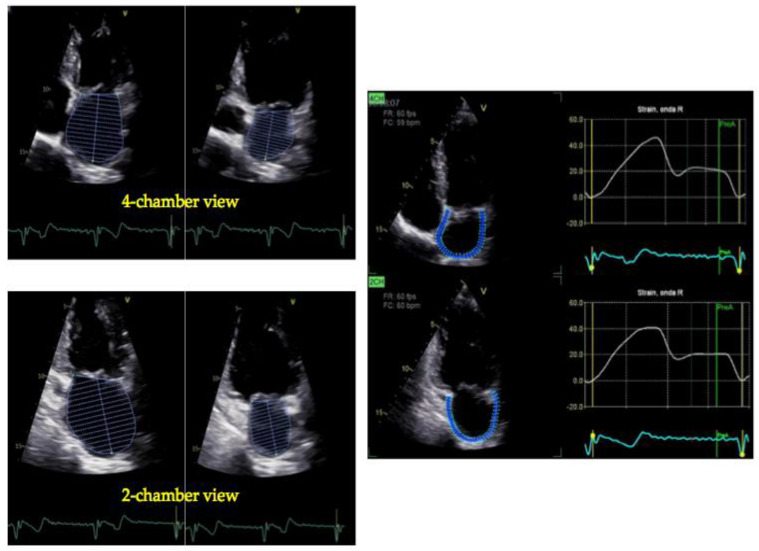
Measurement of LA volume and function in a healthy individual. LA maximal and minimal volumes by four-chamber (upper left) and two-chamber (lower left) views in a 30-year-old healthy man. LAVI is 31 mL/m^2^ and LAEF 59%. In the right panel, the LA function by biplane STE is depicted: the average LA strain is 43%.

**Figure 3:**
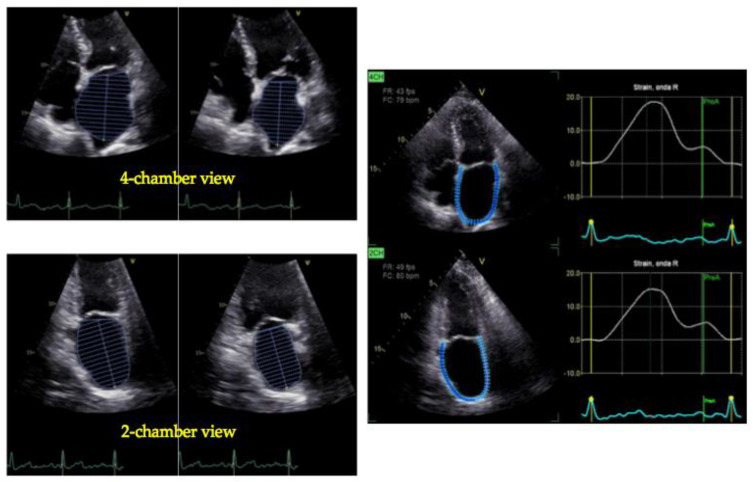
Measurement of LA volume and function in a patient with heart failure. LA maximal and minimal volumes by four-chamber (upper left) and two-chamber (lower left) views in a 56-year-old male with heart failure and mildly reduced ejection fraction. LAVI is 43 mL/m^2^ and LAEF 35%. In the right panel, the LA function by biplane STE is depicted: the average peak LA strain is 19%.

The strain and strain rate using either TDI or STE, measure the extent and the rate of myocardial deformation and may be utilized to evaluate LA function [29,30]. TDI-based strain-rate imaging is challenging, as it is affected by the insonation angle and requires tracking with the motion of the wall to avoid the influence of the translational motion [31]. Despite these limitations, in expert hands feasibility is high, and multiple studies support its relevance in different contexts [19]. The introduction of STE has overcome the difficulties related to TDI-derived technologies [32]. The LA peak longitudinal strain (PALS) can be easily obtained by STE and reliably reflects the LA phasic function. LA strain has demonstrated an association with major CV outcomes in several clinical settings, including all stages of heart failure (HF) [33,34,35]. Normal values for 2D or 3D LA ejection fraction (LAEF) and the peak left atrial longitudinal strain have been explored by multiple studies; those with at least 300 patients are summarized in Table 3. In Figure 2 and Figure 3, calculations of LAEF (biplane 2D) and LA strain in a healthy subject and in a patient with HF with reduced ejection fraction (HFrEF), respectively, are reported.

## 3. Left Atrial Ejection Fraction (LAEF)

Left atrial ejection fraction (LAEF), also known as left atrial emptying fraction, is calculated using the left atrial maximum volume (LAV max) at end-systole and minimum volume (LAV min) at end-diastole, following the formula (LAV max − LAV min)/LAV max. The volumes are measured in both apical four-chamber and apical two-chamber views. The choice of appropriate timing by ECG allows the examination of the global LA function as well as each of its components (Table 1, Figure 1) [44]. LAEF describes atrial functional remodeling more than LA enlargement and can be assessed by different imaging techniques such as 2D and 3D echocardiography, cardiac magnetic resonance (CMR), and computed tomography (CT) (Table 3 shows the normal values of 2D and 3D LAEF). Obtaining accurate and reproducible echocardiographic measures of LA function can be challenging due to multiple patient- and operator-dependent factors. Moreover, an echocardiographic assessment of heart volumes systematically underestimates the chamber size in comparison with CMR and CT [45]. Importantly, LAEF by echocardiography is widely available in multiple clinical scenarios and does not require dedicated software. Similarly to LV myocardial physiology, the Frank–Starling mechanism also applies to the LA myocardium and should be considered for the proper interpretation of LAEF data. For instance, it is well recognized that the longer the duration of AF, the more advanced the pathophysiological alterations, leading to increasing LA dilatation and dysfunction. The prognostic relevance of an echocardiographic LAEF has been assessed by multiple studies encompassing different clinical situations (Table 4), ranging from HF to AF.

Inciardi et al. [46], in participants from the ARIC (Atherosclerosis Risk In Communities) study without prevalent HF, showed that LAEF (and other abnormal LA measures but LAVi) was associated with NT-proBNP levels and with incident HF or death. Moreover, Welles et al. [47], integrating LAEF with the left ventricular outflow-tract velocity time integral, as the LAFI (LAEF xLV outflow-tract velocity time integral]/[LAVI]) showed that any decrease in the LAFI was associated with increased adverse cardiovascular outcomes in patients with coronary arteries disease and preserved EF. In 1951, participants from the 4th Copenhagen City Heart Study, free from atrial fibrillation (AF), functional measures of the LA, including LAEF, were independent predictors of incident AF, particularly in individuals without hypertension [48]. LAEF can also be used to predict postoperative AF, as shown by Darweesh et al. [49], after coronary artery bypass graft (CABG) surgery. Intriguingly, Larsen et al. [50] demonstrated that lower LAEF was associated with the primary endpoint of ischemic stroke, independently of incident AF, in participants from the Copenhagen City Heart Study with no prior history of AF or ischemic stroke. Interestingly, in patients with AF undergoing cardioversion, LAEF, assessed during AF, was significantly larger in the group with sinus rhythm maintenance after 12 months than in the group with AF recurrence [51]. Similarly, higher LAEF was noted in the sinus rhythm compared with the AF recurrence group following ablation at 3 months [52].

**Table 4:** Studies regarding LA function measured by left atrial ejection fraction (LAEF).

Author	Population	Principal Findings
Welles et al., 2012 [47]	855 patients with CAD and EF > 50%	LA dysfunction predicts HF hospitalization
Olsen et al., 2017 [48]	1951 patients without prior AF	Lower LAEF (45 ± 15%) in patients who developed AF
Henriksen et al., 2018 [53]	320 patients hospitalized for AMI	LAEF reduced in response to elevated LV end-diastolic pressure
Walek et al., 2020 [51]	146 patients with persistent AF underwent CVE	LAEF, measured during AF, was superior to predicting SR maintenance after CVE (30.8 ± 8.3 vs. 24.6 ± 10.4%)
Darweesh et al., 2021 [49]	84 patients hospitalized for CABG	LAEF mean of 43% was found in patients developing postoperative AF
Inciardi et al., 2021 [46]	4901 without prevalent HF	LAEF 51.6 ± 11.45% in patients with incident HF or death associated with NT-proBNP levels
Larsen et al., 2022 [50]	1866 patients without known AF or prior ischemic stroke	LAEF decrease was associated with ischemic stroke (mean value 46.4 ± 14.5%)
Khan et al., 2023 [52]	83 patients undergoing AF ablation	Lower LAEF (27.9 ± 9.9% vs. 36.3 ± 10.6%) in patients with AF recurrence after 3 months

Abbreviations: AF, atrial fibrillation; AMI, acute myocardial infarction; CABG, coronary artery bypass graft; CAD, coronary artery disease; CVE, electrical cardioversion; LAEF, left atrial ejection fraction; LV, left ventricle; HF, heart failure; SR, sinus rhythm.

## 4. Peak Atrial Longitudinal Strain

The most studied STE-derived parameter for atrial function is LA strain, which corresponds to the peak atrial longitudinal deformation during the LA reservoir phase. This parameter is automatically calculated by specific software that is available on workstations or last-generation echocardiographic machines [50], using the LA endocardial traces from the four- and two-chamber apical views. However, LA strain curves can be traced either from the start of the P wave or the start of the QRS complex; the QRS as the reference point for the analysis of LA strain is more commonly used. LA strain (Figure 2 and Figure 3) provides essential information about the mechanical function of the LA [54,55] with some limitations, which have been reviewed by Nagueh et al. [56].

Meel et al. [57] showed that LA reservoir function remains stable over decades. A similar finding was reported by Morris et al. [36] using a 2D STE in healthy controls. Moreover, Yoshida et al. [58] identified a significant interaction between gender and LA function. Their study on 414 subjects with paroxysmal and/or persistent AF revealed lower LA systolic strain values in women. LA strain inversely correlates with the fibrosis extent, particularly in persistent AF. Cameli et al. [59] observed a negative correlation between histological fibrosis and LA strain in patients with severe degenerative mitral regurgitation.

Many studies have shown the usefulness of LA strain in predicting elevated LV filling pressures. According to different authors who studied LVFP invasively, LA strain correlates with LVEDP, PCWP, and pre-A pressure when invasively assessed [60,61,62], though it is better in HFrEF patients [63]. It performs better than the LAVI and other commonly guideline-directed echocardiographic measures [62,63,64]. LA strain is considered helpful to grade LVDD [65] and to accurately discriminate between non-cardiac and HF with preserved ejection fraction (HFpEF)-related dyspnea [55], with some potential limitations [56].

LA strain demonstrated good prognostic value in predicting CV events or a new onset of AF in various clinical conditions, including hypertension, diabetes mellitus, and chronic kidney disease [66,67,68]. LA strain is a predictor of cardiovascular events in patients who have suffered acute myocardial infarction. Firstly, according to Madsen et al. [69], a lower LA strain can detect new-onset AF in the post-acute setting. Similarly, Antoni et al. [70] and Ersbøll et al. [71] showed the prognostic role of lower values of LA strain in patients with acute coronary artery disease. More recently, Pastore et al. [72] showed the role of LA strain in predicting postoperative AF in patients undergoing CABG.

Recently, Benfari et al. [34] examined the determinants of left atrial strain and its clinical features across different heart failure stages using data from the EACVI MASCOT registry. The study revealed that LA strain varies significantly with heart failure progression, from the preclinical to clinical stages, and provides valuable insights into cardiac dysfunction. LA strain has been identified as a predictor of new-onset AF in patients with heart failure, thus indicating that LA strain could be a useful marker for monitoring and managing HF patients [73]. Moreover, Park et al. [74] also demonstrated that LA strain is associated with worse clinical outcomes in acute HF, showing its potential as a predictive marker for mortality and adverse events. Aimo et al. [75] explored the diagnostic value of LA strain in cardiac amyloidosis. Their study concluded that LA strain is significantly reduced in patients with amyloid cardiomyopathy and can aid in early diagnosis, providing a non-invasive method to enhance the detection and management of this condition. Essayagh et al. [76] followed for a mean of 21 months 307 consecutive HCM patients, observing that LA strain was strongly associated with the occurrence of cardiac events.

Patients with valvular heart disease (VHD) are at high risk of atrial remodeling and subsequent dysfunction, even before LV alterations [77]. LA strain, mirroring LA function, can identify VHD patients who are at an increased risk of events. According to Thellier et al. [78], both LA strain and diastolic dysfunction are strong predictors of increased mortality in patients with aortic stenosis. Even in mitral regurgitation, lower LA strain values are associated with worse outcomes, as shown in moderate asymptomatic primary mitral regurgitation by Cameli et al. [79] and also in functional mitral regurgitation by Gomes et al. [80], who linked LA strain with all-cause mortality. LA strain has been extensively used in identifying patients at risk of AF. Firstly, a systematic review by Anagnostopoulos et al. [81] showed that lower LA strain can help detect occult AF in patients with cryptogenic stroke. Furthermore, Pagola et al. [82] investigated the predictive value of LA strain and NT-proBNP for AF with a high risk of embolization, finding that reduced LA strain and elevated NT-proBNP levels are strong predictors of embolic events in AF patients. Studies by Alhakak et al. [83] and Hauser et al. [54] showed that LA strain is a significant predictor of both AF and stroke in the general population. Finally, Takagi et al. [84] suggested that, due to its prognostic role, routine LA strain measurements can help tailor treatment strategies for AF patients and improve their clinical outcomes. These and other [85,86,87,88,89,90] studies among major ones regarding LA strain are summarized in Table 5. What is noteworthy is that based on the lower limit of normal LA strain (23%) [36] (Table 3), Morris et al. [88] demonstrated that in patients with LV diastolic dysfunction, elevated LV filling pressures, and a normal LAVI, abnormal LA strain was related to an increased frequency of dyspnea, New York Heart Association functional class III/IV, pulmonary capillary wedge pressure >15 mm Hg, and heart failure hospitalization at 2 years. Moreover, in the same subgroup, abnormal LA strain was the only independent risk for developing HF after adjusting for sex and age. Similar values of LA strain have been correlated with the incidence of AF by others [55,84]. It should also be underscored, however, that a consensus report from the European Association of Cardiovascular Imaging suggested a lower threshold of LA strain (i.e., 18%) for evaluating LV filling pressure both in HFrEF and HFpEF [35].

## 5. Tissue Doppler Imaging and LA Function: A Simplified, Complementary Approach

Measurement of myocardial velocities with tissue Doppler imaging (TDI) has become an integrated part of the assessment of diastolic heart function in clinical echocardiography. Moreover, both systolic (TDI-s’) as well as early (e’) and late (a’) diastolic TDI velocities have been demonstrated to be sensitive markers of impaired cardiac function and prognosis [2,7,91,92]. Importantly, it has been shown that different phases of the TDI curve carry different prognostic information [93].

**Figure 4:**
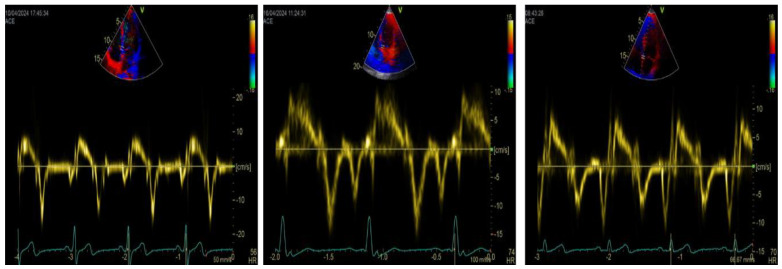
Age and diastolic velocities by tissue Doppler imaging (TDI). Septal TDI traces measured in three healthy male individuals (aged 17, 33, and 62 years) are reported to show the inverse behaviors of early (e’) and late (a’) diastolic TDI velocities across different ages. At 17 years, TDI-e’ and a’ were16 and 6 cm/s, respectively (left panel); at 33 years (mid panel), TDI-e’ and a’ were 11 and 9 cm/s, respectively; at 62 years (right panel), TDI-e’ and a’ were 8 and 11 cm/s, respectively.

TDI-a’ is a fast and accurate marker of atrial systolic function, as it correlates with other measures of LA function. [94,95,96,97,98]. In the general population, TDI-a’ is normally distributed [99]. Differently from early diastolic velocity (TDI-e’), TDI-a’ directly increases with age progressively, resulting in a temporal alignment of LA contraction and late diastolic flow [100,101] (Figure 4).

Finally, all systolic atrioventricular-plane measures correlate with TDI-e’ but not with TDI-a’ [101,102,103,104,105,106,107]. Consistently, with progressing myocardial deterioration, s′ and e′ will decrease early in the disease progression while TDI-a’ will increase, implying compensation by augmentation of atrial function [108].

The relationship between TDI-a’ and LA function has been extensively demonstrated. Invasive studies have shown that TDI-a’ is directly related to LA contractile function, inversely related to LA afterload (i.e., LV end-diastolic pressure), and is affected by myocardial ischemia [109,110,111,112,113]. Moreover, the relationship between TDI-a’ and other echocardiographic indices of LA function, including STE [113], has been demonstrated in different clinical and hemodynamic settings [95,96,97,113,114,115,116]. Interestingly, the echocardiographic parameter that most accurately represented LAEF, obtained by multi-detector computed tomography in 104 consecutive patients scheduled for paroxysmal (AF) ablation, was TDI-a’ [117]. Importantly, there is evidence of an impaired reserve of LA function parameters, including TDI-a’ in patients with HF and preserved ejection fraction (HFpEF) as opposed to hypertensives without HF [100,118,119]. Normal values for TDI-a’ from very large studies are summarized in Table 6 [17,120,121,122,123].

## 6. TDI-a’ and Outcome

TDI-a’ emerges as a valuable prognostic marker, offering insights into LA function and prognosis across various clinical scenarios [107,119,123,124,125,126,127,128,129]. Yamamoto et al. [123] showed that a TDI-a’ cut-off value of <5 cm/s strongly predicted the clinical outcome in HFrEF compared with the clinical, hemodynamic, and other echocardiographic variables. Mogelvang et al. [126] showed that TDI-assessed systolic and both early and late diastolic function provide prognostic information on CV mortality and morbidity independently of and incrementally to the traditional risk factors and biomarkers. Intriguingly, the expected interdependency between early and late diastolic function (Figure 4) was not found in the group who suffered an event during follow-up, indicating that the otherwise expected compensatory mechanism between TDI-e’ and TDI-a’ is impaired in this subset and maybe, in part, responsible for their poor prognosis. Even in patients with ischemic cardiomyopathy and implantable cardioverter-defibrillator, Biering-Sorensen et al. [107] could demonstrate that TDI-a’ predicted future arrhythmic and CV mortality outcomes. In patients with ST-elevation myocardial infarction, Iwahashi et al. [128] showed that TDI-a’, at 24 h, predicted major adverse CV events. Oike et al. [118] demonstrated that HFpEF patients with a TDI-a’ of <7.45 cm/s had a significantly higher risk of total CV- and HF-related events than those with a TDI-a’ of ≥7.45 cm/s.

## 7. TDI-a’ Integrated Echocardiographic Indices

The TDI-a’ velocity has been integrated with other echocardiographic indices. The total atrial conduction time (PA-TDI), defined as the time from the onset of the P wave on an ECG to the peak of TDI-a’ tracing, is a useful marker of atrial remodeling to identify patients at risk for AF, as well as to guide AF management, as extensively reviewed by Müller and colleagues [130]. Moreover, with the progressive development of HF with elevated LV end-diastolic pressure, atrial afterload results in decreased LA contraction—an effect that can be detected by prolonged PA-TDI [131].

**Figure 5:**
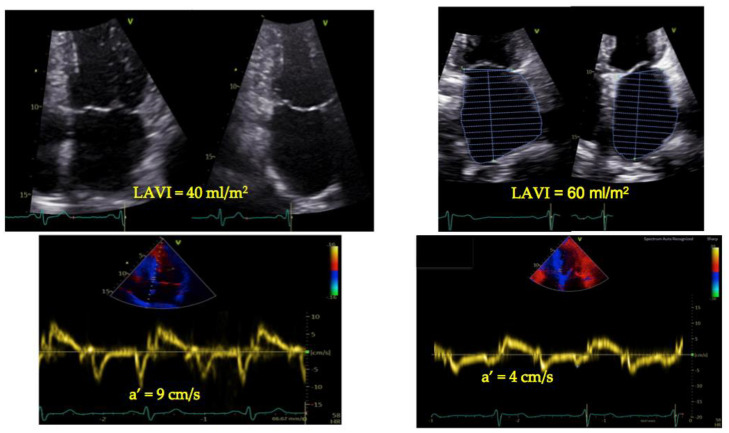
Measurements of left atrial volume index (LAVI) over TDI-a’. LAVI over TDI-a’, renamed LA volumetric/mechanical coupling index (LACI) by Benfari et al. [136], has been shown to mirror the severity of diastolic dysfunction and to correlate with outcomes in different settings. The left panel shows a 58-year-old woman with uncomplicated hypertension, with mildly enlarged LAVI (40 mL/m^2^) and TDI-a’ = 9 cm/s, resulting in LACI = 4.4. The right panel reports a 78-year-old woman with heart failure with preserved ejection fraction due to transthyretin amyloidosis, with severely enlarged LAVI (60 mL/m^2^) and a TDI-a’ = 4 cm/s, reduced in comparison with what is expected for age, resulting in a LACI = 15.

Preclinical atrial dysfunction is characterized by reduced reservoir and conduit function, while atrial contractile function remains normal. As further deterioration of LV compliance occurs, TDI-a’ reduces, and the LAVI progressively increases [132]. This has resulted in considering LAVI/a’ a likely candidate for detecting raised LV end-diastolic pressure (Figure 5).

In 395 patients hospitalized with dyspnea, Park et al. [133] demonstrated that a LAVI/a’ of 4.0 was the best cut-off value to identify advanced DD and was an independent predictor of clinical outcomes. Consistently, Stahrenberg et al. [129] showed that a LAVI/a′ of < 2.3 could effectively rule out paroxysmal AF on 7-day Holter monitoring in 193 consecutive patients with cerebral ischemia and sinus rhythm on presentation. Moreover, the LAVI/a’ enabled a more accurate diagnosis of a history of paroxysmal AF than the conventional parameters in hypertensive patients [134], and LAVI/a′ was related to plasma BNP levels in patients with acute coronary syndrome—being useful for predicting cardiac events in these patients [135].

Interestingly, Setti et al. [13], in 1158 individuals without HF, including 273 healthy individuals, showed that LAVI/a’ mirrored grades of DD and was the single most powerful tool to disentangle the undetermined diastolic function. In particular, by a stepwise regression model, LAVI/a’ was more significantly related to DD grades than a’ alone. Benfari et al. [136], in 4196 patients with HFrEF, showed that LAVI/a’, [renamed the LA volumetric/mechanical coupling index (LACI)], was strongly, independently, and incrementally associated with excess mortality, irrespective of the functional mitral regurgitation grade and in all subsets. Similarly, Essayagh et al. [137], in 4792 patients with floppy mitral valves, reported that the LACI was both associated with worse clinical presentations and incrementally determinant of the outcome. Benfari et al. [138] also demonstrated that the LACI predicted new-onset AF independently from the CHARGE-AF or CHA2DS2-VASc score in the Copenhagen City Heart Study. Finally, Madsen et al. [68] demonstrated that echocardiographic measures of LA function, including LAVI/a’, are independent predictors of AF following acute coronary syndrome, suggesting that evaluation of LA function might improve the prognostic workup, aid in risk stratification for AF, and improve selection for further examinations.

The importance of the LA contribution to cardiac function has become increasingly recognized in clinical practice. The assessment of LA volume by transthoracic echocardiography is an indispensable component of any echocardiographic examination. Dedicated views, appropriate methods, and accurate tracings are all necessary technical skills to obtain an optimal LA volumetric assessment, whose interpretation, however, must consider the multiplicity of factors affecting the LAVI [5,14,16,17]. However, it has become progressively clearer that a morphological evaluation should be integrated with a proper assessment of LA function [7,18]. Historically, Doppler echocardiography, particularly the PVF signal, is utilized to understand LA pressure and function [21]. Regrettably, PVF is relatively underused compared with the transmitral flow to evaluate LA function, though the waveform of PVF enables an assessment of the LA pump, reservoir, and conduit functions [24,134]. Echocardiographic volumetric techniques for assessing LA function have been proposed, with important clinical results achieved [47,48,49,50,51,52], but multiple factors raise concerns about time expenditure and its feasibility in clinical practice. STE represents an undoubted improvement in the assessment of LA function [55,63,72,73,74,75,80,81,82,83,84,85,86,87,88,89,90]. However, using LA function based on STE to predict LV filling pressure [21,56] must take into consideration the presence of either a normal/abnormal LVEF as well as the presence of AF. Even more importantly, in terms of clinical applicability, STE relies on the quality of 2D images and claims dedicated software that only recently became available on echocardiographic machines, thus often needing external workstations. Moreover, issues related to potential vendor dependence raise questions about the usability of the cut-off values obtained by specific software packages [35]. Finally, STE is not available in most non-referral settings, thus limiting its wide implementation in clinical practice.

TDI is a largely available echocardiographic technique and offers a unique possibility for implementing LA function in everyday clinical practice [87,135]. TDI-a’ is correlated with both invasive and non-invasive estimates of LA function [94,95,96,97,98], and its clinical and prognostic significance has been shown in multiple clinical scenarios (Table 7). Interestingly, TDI-a’ has been integrated with other parameters, namely PA-TDI [130,131] and LAVI/a’ (Table 7), with potential interesting clinical and prognostic implications. TDI-a’ thus emerges as a valuable tool in providing a complementary but simple assessment of LA function, which is feasible in a busy clinical setting without the need for post-processing analysis. Of course, TDI-a’ is negatively affected by the insonation angle and unreliability in patients with extensive mitral annular calcification, as is any other TDI measure [139,140,141]. Moreover, the implication of the site of measurement (i.e., septal, lateral, or average) or the influence of major intraventricular conduction disturbances on measurements and interpretation must also be assessed.

Unfortunately, TDI-a’ is often disregarded. This is likely attributable to the fact that the current guidelines support the exclusive utilization of TDI-e’ and E/e’ in the context of the assessment of diastolic function. Moreover, it is not habitually mentioned in the vast majority of research studies devoted to refined, newer technologies such as STE. Furthermore, the determinants of TDI-a’ and other derived indexes, such as LAVI/a’, and their potential in tracking therapeutical interventions in specific clinical settings must be explored. Most importantly, studies comparing a TDI-a’-based evaluation of LA function with well-established STE-based technologies are necessary to ascertain its full significance.

## 8. Conclusions

The current medical evidence underscores the clinical relevance of adding an assessment of LA function to the LAVI to enhance the clinical and prognostic information in the management of CV diseases. Among the methods used to assess LA function by echocardiography, LA strain emerges as the key parameter, supported by the most extensive medical evidence. Additionally, LAEF, using 2D and 3D echocardiography and, to a lesser extent, mitral annular a’ using TDI, are also valuable parameters that provide clinical and prognostic information. Thus, incorporating these parameters, particularly LA strain, into standard LA measurements such as the LAVI in clinical practice provides clinical and prognostic information for better patient management, especially in those with CV risk factors and preserved LVEF.

## Figures and Tables

**Table 1:** Volumetric indices to assess left atrial function.

LA Function	Functional Parameter	Calculation
Global function	LA EF	(LAmax − LAmin)/LAmax
Reservoir function	Expansion index	(LAmax − LAmin)/LAmin
Conduit function	Passive EF	(LAmax − LApreA)/LAmax
Booster pump	Active EF	(LApreA − LAmin)/LApreA

See the manuscript for abbreviations.

**Table 2:** Spectral Doppler indices to assess left atrial function.

Method	Measurement	Clinical Applicability
E/A ratio	E/A ratio, E-VTI/A-VTI	diastolic function
Atrial filling fraction	A-VTI/(E-VTI+A-VTI)	atrial contribution
S/D ratio	S-VTI/D-VTI	relative reservoir to conduit contribution
pulmonary vein atrial reversal wave (Ar)	Ar velocity and duration	atrial contractility
LA ejection force	0.5 × blood density × mitral orifice area × A velocity	LA systolic function
LA kinetic energy	0.5 × blood density × (LAVIpreA-LAVImin) × A velocity	LA work

See the manuscript for abbreviations.

**Table 3:** Normal values of 2D and 3D LA EF and LA strain.

Study	N. Patients	LA EF 2D (%) Mean (IQR) or ± SD	LA EF 3D (%) Mean (IQR) or ± SD	PALS (%) Mean (IQR) or ± SD
Morris et al., 2015 [36]	329	65.8 ± 7.5 LLN 51.1	-	45.5 ± 11.4 LLN 23.1
Pathan et al., 2016 [37]	meta-analysis 40 studies 2542	-	-	39 (95% CI 38–41)
Sugimoto et al., 2018 [38]	371	68.5 (63.2 to 73.2) LLN 48.7 ± 1.9	57.3 (52.4 to 61.9) LLN 41.4 ± 1.1	42.5 (36.1 to 48.0) LLN 26.1
Takeuchi et al., 2019 [39]	313	-	M 48 ± 9 F 48 ± 11	-
Sun et al., 2020 [40]	324	-	-	35.9 ± 10.6
Singh et al., 2021 [15]	1765	65.7 ± 8.4	62.2 ± 7.7	42.1 ± 10.0
Nielsen et al., 2021 [41]	1641	-	-	39.4 (33.2–46.6)
Wang et al., 2024 [42]	783	-	M 57.3 ± 5.7 F 57.5 ± 6.4 LLN M 46; F 44	-
Yafasov et al., 2024 [43]	979	-	61 ± 6	31.2 ± 6.3

Based on the way data were reported by single studies, numbers are mean (IQR) or ± SD. Moreover, when available, LLNs are included. Abbreviations: CI: confidence interval; F: females; IQR: interquartile ranges; LLN: lower limit of normal; M: males; PALS: peak left atrial longitudinal strain. SD: standard deviation.

**Table 5:** Studies regarding LA function measured by LA strain.

Author	Population	Principal Findings Regarding LA Function, Measured by PALS
Cameli et al., 2012 [85]	312 adults in SR	PALS < 18.8% is associated with the development of the first CV event (sens. 78.2%, spec 85.2%, AUC 0.83)
Freed et al., 2016 [86]	308 HFpEF longitudinally followed	1-SD decrease in PALS is associated with composite outcome of hospitalization or death (HR 1.54)
Santos et al., 2016 [87]	357 HFpEF enrolled in the TOPCAT study	Unit reduction in PALS is associated with an increased risk of CV events
Morris et al., 2017 [88]	517 patients in SR and risk factors for LVDD	Adding LA strain (cut-off < 23%) to LAVI significantly improves the detection of LVDD in indeterminate LV diastolic function
Cameli et al., 2019 [89]	276 patients with asymptomatic moderate MR	PALS < 35% is associated with the development of CV events (AUC 0.87)
Park et al., 2020 [73]	2461 patients with AHF and SR	PALS < 18% predicts new-onset AF at 5 years (AUC 0.53)
Nielsen et al., 2020 [90]	Meta-analysis of 1025 patients undergoing RFA for AF	PALS significantly predicts AF recurrence (multivariate OR 1.16 CI95% [1.09–1.24], *p* < 0.001, per 1% decrease)
Park et al., 2021 [74]	3818 AHF patients	PALS is a significant predictor of events regardless of HF phenotype (multivariate PALS < 8.8%: HR 1.637, *p* = 0.001, PALS 8.8-16.5%: HR 1.416, *p* = 0.004)
Inoue et al., 2021 [63]	322 patients with CV disease of different etiologies	PALS < 18.0% predicts elevated LV falling pressure
Pagola et al., 2021 [82]	253 patients with cryptogenic stroke followed for 2 years	PALS < 25.3% predicted HpAF with a sensitivity of 70% and specificity of 60% (AUC 0.73)
Hauser et al., 2021 [55]	3590 general population in SR	Patients with PALS < 23% have a 6.8 increased risk of developing AF; with multivariate, PALS remained an independent predictor of AF [HR 1.05, 95% CI (1.03–1.07), *p* < 0.001, per 1% decrease]
Alhakak et al., 2022 [83]	400 general population in SR	PALS independently predicts AF in participants < 65 years (HR 1.46; 95% CI (1.06–2.02), *p* = 0.021, per 5% decrease)
Aimo et al., 2022 [75]	423 patients screened for cardiac amyloidosis	PALS < 6.65% gives 3.6-fold risk of cardiac amyloidosis
Thellier et al., 2023 [78]	387 patients with severe aortic stenosis	PALS < 14% improves mortality risk stratification over diastolic dysfunction grades
Madsen et al., 2023 [69]	381 patients with ACS	With univariate analysis, PALS predicts new onset of AF (HR: 1.05, *p* < 0.01, per 1% decrease)
Gomes et al., 2023 [80]	307 HFrEF with functional MR	PALS predicts all-cause mortality with HR: 1.05 per each 1% decrease
Takagi et al., 2023 [84]	335 patients	PALS < 22% predicts new-onset AF (AUC: 0.89)
Anagnostopoulos et al., 2023 [81]	Meta-analysis of 2081 patients with cryptogenic stroke	PALS < 20% presents 71% sensitivity and 71% specificity for the diagnosis of occult AF
Pastore et al., 2024 [72]	310 patients undergoing isolated CABG	multivariate analysis, PALS < 28% carries a 3.6-fold higher risk of postoperative AF

Based on the way data were reported by single studies. Abbreviations: ACS: acute coronary syndrome; AHF: acute heart failure; AUC: area under curve; CABG: coronary artery bypass graft; CI: confidence interval; CV: cardiovascular; HFeEF: HF with reduced ejection fraction; HFpEF: HF with preserved ejection fraction; HpAF: paroxysmal atrial fibrillation with high risk of embolization (AF episodes > 5 h); HR: hazard ratio; LAVI: left atrial volume index; LVDD: left ventricular diastolic dysfunction; MR: mitral regurgitation; PALS: peak atrial longitudinal strain; RFA: radiofrequency ablation; SD: standard deviation; SR: sinus rhythm.

**Table 6:** Normal values of TDI-a’.

Study	N. Patients	Age (Years)	TDI-a’ Septal Mean (IQR) or ± SD	TDI-a’ Lateral Mean (IQR) or ± SD	TDI-a’ Average Mean (IQR) or ± SD
Daimon et al., 2008 [120]	M 383 F 317	20–79	M 9.2 ± 2.1 F 8.2 ± 2.4	M 9.0 ± 3.0 F 8.3 ± 2.7	-
Ruohonen et al., 2015 [121]	M 444 F 635	34–49	M 12.9 ± 2.2 * F 12.4 ± 2.1 *	M 14.0 ± 3.0 * F 13.7 ± 3.0 *	-
Caballero et al., 2015 [122]	444	20–75	9.4 ± 2.0 IQR 8.0–11.0	9.2 ± 2.7 IQR 7.0–11.0	11.9 ± 3.3 IQR 9.25–14.0
Yao et al., 2016 [123]	M 678 F 716	18–79	M 9.4 ± 2.1 F 8.9 ± 2.1	M 9.9 ± 2.6 F 9.8 ± 2.8	M 9.6 ± 2.1 F 9.3 + 2.1
Ballo et al., 2017 [17]	282	7–84	-	-	10.1 ± 2.4

Based on the way data were reported by single studies, numbers are mean (IQR) or ± SD unless otherwise specified. Abbreviations: F: females; IQR: interquartile ranges; M: males, SD: standard deviation. * median.

**Table 7:** Summary of major studies assessing the relevance of TDI-a’ or LAVI/a’ (LACI).

Study	Population	Study Design	Cut-Off	Aims/ End-Point
Studies with TDI-a’				
Yamamoto et al., 2003 [124]	HFrEF	Prospective	TDI-a’ ≤ 5 cm/s	Cardiac mortality
Wang et al., 2003 [125]	Patients with and without cardiovascular disease	Retrospective	TDI-a’ < 4 cm/s	Cardiac death
Mogelvang et al., 2015 [127]	General population	Prospective	HR 1.17 for 1 cm/s decrease in TDI-a’	Acute myocardial infarction, HF, CV death
Biering-Sorensen et al., 2016 [107]	Ischemic cardiomyopathy	Prospective	Multivariate TDI-a’ HR 1.25	Arrhythmic events, CV death
Oike et al., 2020 [119]	HFpEF	Prospective	TDI-a’ > 7.45 cm/s	CV and HF events
Iwahashi et al., 2021 [129]	ST-elevation myocardial infarction	Prospective	TDI-a’ < 9.4 cm/s at 24 h	Major adverse CV events
**Studies with LAVI/a’ (LACI)**				
Stahrenberg et al., 2011 [128]	Cerebral ischemia	Prospective	LAVI/a’ < 2.5 to rule out AF	AF onset
Park et al., 2011 [134]	Patients with dyspnea (NYHA II-IV)	Prospective	LAVI/a’ ≥ 4 higher incidence of outcome	Cardiac death/ rehospitalization HF
Benfari et al., 2021 [137]	HFrEF in sinus rhythm with MR	Prospective	LAVI/a’ ≥ 6 excess mortality	Survival
Essayagh et al., 2022 [138]	Patients with floppy mitral valve in sinus rhythm	Prospective	LAVI/a’ ≥ 5 excess mortality	Survival
Benfari et al., 2023 [139]	General population	Prospective	LAVI/a’ > 3.9	AF onset independently from CHARGE-AF and CHA2DS2-Vasc

See the manuscript for abbreviations.
